# Amyloid‐beta subspecies in individuals with Down Syndrome, sporadic Alzheimer's disease (sAD) and PS1 mutations ‐ comparison using IP‐Mass Spectrometry of plasma

**DOI:** 10.1002/alz70856_107136

**Published:** 2026-01-08

**Authors:** Andre Strydom, Asaad Baksh, Marie‐Claude Potier, Laia Montoliu‐Gaya, Mina Idris, Leda A Bianchi, Nicholas Ashton, Sarah Pape, Henrik Zetterberg

**Affiliations:** ^1^ King's College London, London, United Kingdom; ^2^ South London and the Maudsley NHS Tust, London, United Kingdom; ^3^ The LonDownS consortium, London, United Kingdom; ^4^ Institute of Psychiatry, Psychology and Neuroscience, King's College London, London, United Kingdom; ^5^ ICM Institut du Cerveau et de la Moelle épinière, CNRS UMR7225, INSERM U1127, UPMC, Hôpital de la Pitié‐Salpêtrière, 47 Bd de l'Hôpital, F‐75013 Paris, France, Paris, France; ^6^ Institute of Neuroscienace and Physiology, University of Gothenburg, Mölndal, Västra Götaland, Sweden; ^7^ Department of Psychiatry and Neurochemistry, Institute of Neuroscience and Physiology, The Sahlgrenska Academy, University of Gothenburg, Mölndal, Sweden; ^8^ UCL Institute of Neurology, London, United Kingdom; ^9^ Institute of Neuroscience and Physiology, The Sahlgrenska Academy at University of Gothenburg, Mölndal, Sweden

## Abstract

**Background:**

Down Syndrome (DS) individuals develop Alzheimer's Disease (AD) neuropathology by the age of 40 due to the triplication of Chromosome 21 and the amyloid precursor protein gene. This genetic alteration leads to an overproduction of Aß, resulting in amyloid accumulation. However, triplication of other Chr21 genes may be involved in AD pathogenesis in DS, and affect amyloid processing and alter subspecies production. This study compares plasma Aß subspecies using mass spectrometry (MS) in DS, sAD and PS1 mutations.

**Method:**

Plasma from adults with DS who were cognitively stable (CS‐DS; *n* = 46; mean age 33.5), cognitive stable older adults from the general population (CS; n = 42; mean age 71.7), DS deemed prodromal or with AD (DS pAD/AD; n = 25; mean age 53.5), early onset sporadic pAD/AD (*n* = 27; mean age 63.9) and with presenilin 1 mutations (*n* = 6; mean age 50.5) were analysed using IP‐MS for Aß 1‐37, 1‐38, 1‐40, and 1‐42. In those with DS, Simoa assays for Aß 40 and 42, neurofilament light (Nfl), glial fibrillary protein (GFAP, pTau181 and pTau231 were also obtained, and an Event‐Based Model (EBM) was used to determine the sequence of changes associated with AD.

**Result:**

DS individuals had consistently higher levels of Aß1‐37, 1‐38, 1‐40, and 1‐42 compared to CS general population adults, sporadic pAD/AD, and those with PS1 mutations. DS pAD/ AD had increased Aß ratios for 1‐ 40/1‐37 and 1‐37/ 1‐38 compared to sporadic pAD/AD, but similar ratios when compared to those with PS1 mutations. Age‐related patterns of changes in Aβ1‐37 and 1‐38 subspecies in DS pAD/AD differed compared to sporadic pAD/AD, but changes in Aβ 1‐40 and 1‐42 levels were similar. APOE ε4 was associated with Ab 1‐37 levels in DS. Changes Aβ1‐42/1‐38, 1‐42/1‐37, and 1‐42/1‐40 were early events followed by changes in NfL, GFAP, pTau 181 and pTau 231.

**Conclusion:**

All Aß subspecies are increased in DS compared to sAD, with higher 1‐40/1‐37 ratios and different age associated changes. Measurement of Aβ subspecies with IP‐MS may help to identify early AD in DS. Further work could determine the nature of differences in subspecies compared to sAD.

